# A study protocol testing pre-exposure dose and compound pre-exposure on the mechanisms of latent inhibition of dental fear

**DOI:** 10.1186/s40359-024-01527-w

**Published:** 2024-01-18

**Authors:** Andrew L. Geers, Laura D. Seligman, Keenan A. Pituch, Ben Colagiuri, Hilary A. Marusak, Christine A. Rabinak, Natalie Turner, Sena L. Al-Ado, Michael Nedley

**Affiliations:** 1 Department of Psychology, University of Toledo, Toledo, OH 43606 USA; 2 Department of Psychological Science, University of Texas Rio Grande Valley, Edinburg, TX USA; 3 Edson College of Nursing and Health Innovation, Arizona State University, Phoenix, AZ USA; 4 Department of Psychology, University of Sydney, Sydney, Australia; 5 Department of Psychiatry and Behavioral Neurosciences, School of Medicine, Wayne State University, Detroit, MI USA; 6 Department of Pharmacy Practice, Wayne State University, Detroit, MI USA; 7 Department of Dentistry, University of Toledo College of Medicine and Life Sciences, Toledo, OH USA

**Keywords:** Dental phobia, Latent inhibition, Fear learning, Pre-exposure, Pain sensitivity, virtual reality, Eye tracking

## Abstract

**Background:**

Dental stimuli can evoke fear after being paired - or conditioned - with aversive outcomes (e.g., pain). Pre-exposing the stimuli before conditioning can impair dental fear learning via a phenomenon known as latent inhibition. Theory suggests changes in expected relevance and attention are two mechanisms responsible for latent inhibition. In the proposed research, we test whether pre-exposure dose and degree of pre-exposure novelty potentiate changes in expected relevance and attention to a pre-exposed stimulus. We also assess if the manipulations alter latent inhibition and explore the possible moderating role of individual differences in pain sensitivity.

**Methods:**

Participants will be healthy individuals across a wide range of ages (6 to 35 years), from two study sites. Participants will undergo pre-exposure and conditioning followed by both a short-term and long-term test of learning, all in a novel virtual reality environment. The unconditioned stimulus will be a brief pressurized puff of air to a maxillary anterior tooth. Pre-exposure dose (low vs. high) and pre-exposure novelty (element stimulus vs. compound stimuli) will be between-subject factors, with stimulus type (pre-exposed to-be conditioned stimulus, a non-pre-exposed conditioned stimulus, and an unpaired control stimulus) and trial as within-subject factors. Pain sensitivity will be measured through self-report and a cold pressor test. It is hypothesized that a larger dose of pre-exposure and compound pre-exposure will potentiate the engagement of the target mechanisms and thereby result in greater latent inhibition in the form of reduced fear learning. Further, it is hypothesized that larger effects will be observed in participants with greater baseline pain sensitivity.

**Discussion:**

The proposed study will test whether pre-exposure dose and compound stimulus presentation change expected relevance and attention to the pre-exposed stimulus, and thereby enhance latent inhibition of dental fear. If found, the results will add to our theoretical understanding of the latent inhibition of dental fear and inform future interventions for dental phobia prevention.

Research suggests the prevalence of dental anxiety in the United States for adults is approximately 15%, with estimates for youth ranging between 5% and 20% [1–3]. Dental anxiety remains a substantial barrier to recommended oral health care; it is associated with fewer regular dental visits [4, 5], avoidance of dentist advocated treatment [6], and a greater likelihood of missing, diseased, and filled teeth [7]. Poor oral health, which is increased by dental fear and anxiety, has been linked with adverse health consequences beyond the oral cavity, such as heart disease, stroke, and diabetes [8, 9]. Dental anxiety disproportionately affects marginalized individuals in the United States, including Hispanic and African American populations [10, 11].

Consistent with the broader experimental literature on fear and anxiety, direct associative conditioning appears central to the probability of developing dental fear [2]. For example, studies [12–14] report that dental anxiety is higher for individuals who underwent distressing and painful dental experiences early in their dental history. Townend et al. [15] similarly found that youth who were diagnosed with dental anxiety had experienced more early traumatic dental office visits than youth not diagnosed with dental anxiety.

Naturalistic studies suggest pre-exposure to dental stimuli and contexts prior to direct conditioning can impair the development of dental fear. For example, in studies by Davey [12] and De Jongh et al. [13], the experience of non-fearful dental events prior to potentially fearful or distressing dental events, was related to a lower likelihood of dental anxiety. These findings are consistent with research on the learning phenomenon of latent inhibition (LI). LI occurs when previous passive exposure to a stimulus impairs one’s ability of acquiring or expressing a new association with that stimulus [16]. For example, presenting a stimulus alone prior to conditioning trials in which it is paired with a painful shock reduces learning from the conditioning trials [17].

LI offers a novel opportunity for reducing the probability of dental fear and phobia and improving oral health outcomes. However, the mechanisms underlying LI of dental fear remain unknown. Understanding the mechanism of LI is essential for constructing effective LI interventions that produce effects of the magnitude that yield clinically meaningful outcomes. Hall and Rodriguez [18] have developed a model of the mechanisms of LI that has successfully explained many human and animal examples of LI in other areas. The starting point of this model is that novel stimuli have high informational value and thereby attract attention. When pre-exposure of a novel stimulus occurs; that is, the stimulus is presented without an unconditioned stimulus (UCS), the heightened attention results in the learning of a “stimulus → no event” association. Subsequent passive presentations of this stimulus will lead to a decrease in expected relevance (e.g., prediction errors), as the individual is receiving confirmation for the prediction of the stimulus → no event relationship. Over time, learning that this pre-exposed stimulus is not a signal with informational value leads to a withdrawal of attention to it. Finally, if the stimulus is subsequently paired with an UCS, the lack of attention reduces the associability potential of the stimulus. That is, it results in LI.

Research evidence aligns with the predictions of the Hall and Rodríguez [18] model. For example, blocking of endogenous opioids, which encode expectations [19–21] and help direct attention to relevant stimuli [22], eliminate pre-exposure induced LI. Also, participants receiving pre-exposure to a stimulus prior to pairing that stimulus with a UCS are subsequently less likely to accurately recall the pre-exposed stimulus and are less likely to identify the association between the pre-exposed stimulus and the UCS [23, 24].

Based on the Hall and Rodríguez [18] model, it can be anticipated that certain changes to the pre-exposure conditions should increase engagement of the target mechanisms of expected relevance and attention, and in doing so, strengthen LI. Prior studies support this possibility. For example, animal and human studies find that increasing the amount of pre-exposure, i.e., the number of pre-exposure trials, results in greater changes in attention to the pre-exposed stimulus and also to greater LI [17, 25]. As such, it may be surmised that increasing *pre-exposure dose* will potentiate LI of dental fear by reducing expected relevance and attention to the pre-exposed stimulus, in line with the Hall and Rodríguez model. Another change to the condition of pre-exposure that may alter the mechanisms of LI is *pre-exposure stimulus novelty*. Following from the Hall and Rodríguez model, LI should be strengthened when changes in the environment are introduced that renew the novelty of the pre-exposed stimulus, thus allowing for additional learning of the existing stimulus → no event association. Prior research shows one way to accomplish this is with compound conditioning. In compound conditioning, initial pre-exposure trials are followed with additional trials that have a novel stimulus presented conjointly with the pre-exposed stimulus [26, 27]. Compound pre-exposure should increase LI because the learning of the stimulus → no event association in pre-exposure faces diminishing returns. Introducing a novel stimulus with the pre-exposed stimulus should reactivate the stimulus → no event association and result in greater capacity to learn about the pre-exposed stimulus again. If the target pre-exposed stimulus is still paired with no adverse outcome; the learning of the stimulus → no event association should become stronger. In other words, compound pre-exposure should result in greater engagement of the target mechanisms as the individual is now more confident that the pre-exposed stimulus is not an important signal of anything, and this should result in greater LI of fear learning.

Finally, studies have also found that conditions that increase attention to a stimulus or increase early expectations of stimulus → important outcome predictions during pre-exposure (i.e., expected relevance) lead to greater LI [28]. Individual differences that promote increased attention and expected relevance should lead to potentiation of the LI effect. In the context of fear conditioning with an averse UCS, pain sensitivity is such a characteristic [29].

## Study aim and hypotheses

The aim of the proposed study is to test if a larger pre-exposure dose and compound stimulus presentation potentiates changes in expected relevance and attention to the pre-exposed stimulus, and LI of dental fear. We will also examine whether these effects are larger in individuals with higher level of pain sensitivity.

### Hypothesis 1

A larger dose of pre-exposure, compared to a smaller dose, will (a) increase engagement of the target mechanisms (expected relevance and attention), and (b) strengthen the LI of conditioned fear acquisition, recall, and retention.

### Hypothesis 2

Compared to pre-exposure with only the target stimulus, compound pre-exposure will (a) lead to stronger engagement of the target mechanisms of expected relevance and attention, and (b) strengthen the LI of conditioned fear acquisition, recall, and retention.

### Hypothesis 3

Higher pain sensitivity scores will be associated with increased engagement of the target mechanisms of expected relevance and attention.

## Methods

### Participants

This is a multisite study, with participants enrolled at universities in south Texas and northwest Ohio. For the United States, the region surrounding the Texas site has a larger than average Hispanic population [30], whereas the region surrounding the Ohio site has a substantial African American population [30]. The sites are therefore expected to provide opportunity to recruit Hispanic and African American participants– populations that are of greater risk for dental anxiety and poor oral health [10, 11].

Community volunteers between the age of 6 and 35 will be recruited using strategies such as a study Facebook page, ads posted in social media, newspapers, email announcements, and flyers at community sites. The sample size of 180 participants was determined by an a priori power analysis. Table 1 provides the inclusion/exclusion eligibility criteria for participation. The study will consist of two in-person visits, scheduled approximately 1 week apart, with participant remuneration of a $50 gift card per visit. The Institutional Review Board of the primary institution approved the research.

**Table 1:** Inclusion and exclusion criteria

***Inclusion Criteria***
Be between the ages of 6 and 35 years old
At least 2 of their maxillary anterior 6 teeth present
All of their maxillary anterior 6 teeth are stable
All of their maxillary anterior 6 teeth are free of cavities
All of their maxillary anterior 6 teeth are free of hypersensitivity to pain or pressure
Able to read, write, and converse in English (English or Spanish at the Texas site)
Willing and able to provide a signed and dated informed consent/assent form to participate
Willing and available to comply with all study procedures and available for the duration of the study
***Exclusion Criteria***
Currently have glasses or use contact lenses with a vision correction of ≥ ± 6
Are colorblind
Current injury (including fractures, open cuts, or sores) on their dominant hand
Fixed dental or orthodontic appliance that would interference with us creating a mouthpiece
Unwilling to remove facial cosmetics or attend sessions without facial cosmetics on
Currently on any anti-anxiety or anti-depressant medications
Currently have an inner ear infection
Living in the same household or an immediate family member of an enrolled participant
Currently have a cardiovascular disorder or a pacemaker
Currently have a seizure disorder or epilepsy
History of frostbite or sensitivity to extreme cold
Bleeding disorder or take blood thinners
Vasospastic disorder such as Raynaud’s syndrome or Raynaud’s disease
Gastrointestinal or vestibular disorders that may elevate susceptibility to nausea and dizziness such as Meniere’s disease, hyperemesis gravidarum or severe migraines
Developmental, behavioral, or sensory disorder that would increase discomfort or ability to complete tasks during the study
Prior negative outcome with VR simulation
Diagnosis of Temporomandibular Disorder along with a history of exacerbation of symptoms resulting from routine dental procedures
Medical condition that requires them to avoid mild stress
Any medical condition that elevates risk of falls, dizziness, nausea, or causes a vasovagal reaction
Any other general or oral health concerns

### Experimental design

The study will use a mixed design, with two within-subject independent variables and two between-subjects independent variables, and one measured continuous moderator variable. One within-subjects variable is stimulus type [stimulus type: pre-exposed to-be conditioned stimulus (CS^+^ _P_), non-pre-exposed to-be conditioned stimulus (CS^+^ _NP_), and an unpaired control stimulus (CS^−^)]. The second within-subjects variable is time [trial number]. One of the two-level between-subjects independent variables is pre-exposure dose [12 trials vs. 24 trials]. The second two-level between-subjects independent variable is pre-exposure novelty [single stimulus vs. compound stimuli]. Participants will be randomly assigned to one of the four between-subject conditions using the randomization feature in the software Research Electronic Data Capture (REDCap; 31, 32). Randomization will be done in session to obtain allocation concealment [33]. The continuous moderator variables are two individual-difference measures of pain sensitivity.

### Study timeline

#### Screening

To determine eligibility, research staff will schedule a phone or video call with interested individuals. Adults will complete screening and screening consent for themselves, whereas screening and screening consent will be done with a parent or legal guardian for children and adolescents. After consent, individuals will be screened on the inclusion and exclusion criteria (Table 1). Those interested and meeting criteria will be scheduled for two study visits, approximately one week apart (min = 7 days). The first visit will take approximately 90 min and the second approximately 30 min.

#### Visit 1

The first visit will begin with a confirmation of eligibility and completion of the informed consent and assent (if applicable). Parent or legal authorized guardian will provide permission for child and adolescent participants. Participants will then complete the individual difference measures described in the Moderator section below. Next, to prepare for the experimental task, participants will have a personalized dental mouthpiece made, electrodermal activity electrodes placed on their hand, and instructions for the virtual reality task will be provided. Participants will be randomized to condition and then will complete the experimental task. During the experimental task, the within-subject and between-subject independent variables will be manipulated, and pre-exposure and fear conditioning procedures will be conducted. Fear recall will be assessed immediately after fear conditioning in Visit 1.

### Visit 2

A minimum of 7 days later, participants will return and fear retention will be assessed.

### The experimental task

The experimental task and procedures are described in detail by Seligman et al. [29]. The experimental task takes place in a novel immersive virtual reality environment in which participants traverse a rich alien landscape, programmed using Vizard software [34]. Participants will interact with this environment using HTC Vive Pro headset and controllers [35]. A benefit of using virtual reality is that it provides a method for experimentally controlling pre-exposure (i.e., stimulus novelty) while allowing for the direct assessment of mediating and dependent variables (i.e., eye movements, approach/avoidance behavior). The task will be described to participants as a game in which their goal is to collect fuel canisters on an unfamiliar alien planet so they can power their ship and get back home. Participants will be told that the alien planet is not like Earth and the inhabitants and events may follow different rules. They will additionally be instructed that some of the alien interactions may be somewhat uncomfortable. These instructions are designed to trigger schemas related to pain sensitivity, paralleling experiences at a dental visit, while providing a reason for the mouthpiece they will wear throughout the experimental task.

### Procedures

Participants will experience the virtual reality “game” seamlessly each visit however, the task has four discrete phases: pre-exposure, conditioning, and learning recall phases, all occurring in Visit 1, and a learning retention phase, which occurs in Visit 2. Next, we describe the four phases, explaining the trials in each phase.

Trials in the first phase, the *pre-exposure phase*, will begin with a minimum of six seconds in which the participant is free to navigate around the planet surface to collect fuel concealed in the landscape. Following the initial trial segment, the CS^+^ _P_ is automatically presented, accompanied by a fuel canister, at the first opportunity. The CS^+^ _P_ will be an alien stimulus that displays small vertical movements and associated individualized noises. The CS^+^ _P_ and the fuel canister will appear at a fixed distance from the participant and will be present for the remaining six seconds of the trial. Thus, each trial consists of a six second segment without the CS^+^ _P_, followed by a six second segment with the CS^+^ _P_. During pre-exposure, the CS^+^ _P_ is never accompanied by the UCS.

It is during the *pre-exposure phase* that the two between-subject independent variables are manipulated. First, the number of pre-exposure trials constitute the *pre-exposure dose* independent variable and will be either 12 trials (low dose) or 24 trials (high dose), based on random assignment. Second, for participants in the *single stimulus pre-exposure type condition*, the CS^+^ _P_ will always present in the environment without another alien stimulus. For participants in the *compound stimulus pre-exposure type condition*, a second novel alien stimulus will be immediately adjacent to the CS^+^ _P_ with the presentation period identical to that of the CS^+^ _P_ on the second half of the pre-exposure trials: the final 6 trials for those in the low dose condition, and the final 12 trials for those in the high dose condition.

The *conditioning phase* will consist of 36 trials (12 CS^+^ _P_ trials, 12 CS^+^ _NP_, and 12 CS^-^ trials) occurring in a pseudo-random order. The structure of the conditioning trials is the same as the pre-exposure trials, with the key exception that 75% of CS^+^ _P_ trials, and 75% of CS^+^ _NP_ co-occur with a dental startle UCS (see stimulus material section below). The dental startle UCS will be delivered through a fitted mouthpiece during the last 100ms of the trial with the alien and startle stimulus co-terminating. The dental startle stimulus will never be delivered on CS^-^ trials.

Immediately following the conditioning trials will be the fear *recall phase*, which is a series of 12 trials (4 CS^+^ _P_ trials, 4 CS^+^ _NP_, and 4 CS^-^ trials). The structure of the trials in this phase will be the same as in the prior two phases. During this phase, the dental startle UCS will not accompany any of the alien stimuli.

The trials in the fear *retention phase*, occurring at least 7 days later, are the same as those in the fear recall phase.

### Stimulus material

#### Alien images

Novel alien stimuli were developed and pilot tested with children, adolescents, and adults to obtain stimuli that were perceived as relatively neutral, yet distinct. To reduce any potential influence of specific alien stimuli on study outcomes, three alien stimuli will be counterbalanced for the CS^+^ _P_, CS^+^ _NP_, and CS^-^. The compound alien stimulus will be held constant.

#### Dental startle UCS

The UCS is this study will be the same employed by Seligman et al. [29], a 60 psi air puff delivered to a maxillary anterior tooth for 100ms through an personalized dental mouthpiece, fabricated with 3 M™ STD Vinyl Polysiloxane Express Putty. The air puff will reach the mouthpiece through 3/16 inch tubing connected to a California Air Tools 8010 Steel Tank Air Compressor via an AIRSTIM device (San Diego Instruments, San Diego, California, USA). Prior to the experimental task, participants will be informed that the mouthpiece will allow them to experience different sensations on the alien planet. Participants will wear the mouthpiece throughout the experimental task.

### Study measures

#### Mediating variable measures

*Expected relevance* will be measured after the onset of the CS^+^ _P_, CS^+^ _NP_, or CS^-^ but prior to the onset of the UCS. To obtain ratings of the probability of a negative event occurring, participants will be presented the question “How likely is it something bad is about to happen?” and provide a response on a 10-point scale. The question will appear in front of participants in the virtual environment and ratings will be made using a hand controller.

*Attention* will be assessed with software in the Vive Pro headset that tracks eye movements to create a measure of dwell time.

#### Fear learning measures

Fear learning will be assessed with subjective, physiological, and behavioral indicators. For the subjective indicator, participants will periodically rate their level of relaxation/anxiety when the CS^+^ _P_, CS^+^ _NP_, and CS^−^ are presented. The subjective fear question will be, “How relaxed do you feel?”, with responses provided on a 10-point scale. Skin conductance responses (SCRs) to the CS^+^ _P_, CS^+^ _NP_, and CS^−^ will serve as a physiological indicator of fear learning. To measure SCRs, we will use Biopac MP160 with a wireless BioNomadix module transmitter (Biopac Systems, California, USA.) and Biopac’s Acknowledge 5.0 software (Biopac Systems, California, USA.). Recordings will be made with two Ag–AgCl electrodermal conductance electrodes with Isotonic gel placed on the middle phalanges of the non-dominant hand pinky and ring fingers. SCRs will be analyzed based on prior work [36]. Finally, two variables will be acquired during the trials to provide behavioral indices of fear learning. The first is the shortest distance between the participant and the CS^+^ _P_, CS^+^ _NP_, and CS^−^, which will be recorded on each trial. Second, we will also record whether or not the participant approached the CS^+^ _P_, CS^+^ _NP_, and CS^−^ and obtained the “fuel cell” they appeared with [29].

#### Moderator variable measures

*Pain sensitivity* will be assessed with a self-report instrument- the Fear of Pain Questionnaire III [37] in individuals 18 years of age or older and the Fear of Pain Questionnaire, Child report in individuals from 6 to 17 years old [38]. The cold pressor test will be used to provide a behavioral index of pain sensitivity.

### Statistical analysis plan

SPSS software will be used to address Hypotheses 1a, 2a, and 3 described below. Mplus software will be used to address the remaining hypotheses. Note that throughout the analyses, an interaction between the two between-subjects factors is not of primary interest [25]. Rather, we anticipate that a particular combination of the groups, or the focal group, (i.e., high dose and compound condition) will have more favorable responses than each of the other groups. Consequently, we treat these between-subjects factors as one factor with four groups. Note that representing the four cells of the between-subjects factors in this way yields the same fit as a full between-subjects factorial model and readily enables us to assess the comparisons between the focal and other groups.

#### Hypothesis

1a: A larger dose of pre-exposure, compared to a smaller dose, will increase engagement of the target mechanisms (expected relevance and attention). Hypothesis 2a: Compared to pre-exposure with only the target stimulus, compound pre-exposure will lead to stronger engagement of the target mechanisms of expected relevance and attention.

For Hypotheses 1a and 2a, changes expected in the candidate mediators should first be observed during the pre-exposure phase. To test these hypotheses during this phase, a two factor repeated measures MANOVA with one-between (pre-exposure group) and one-way within-subjects (trial) factor will be conducted separately for the variables expected relevance and attention. A significant group × trial interaction indicates that mean responses across trials is not the same for each group. If the interaction is present, follow-up analyses will focus on examining the plots of the mean change for each group and testing the degree to which the mean change from first to last trial differs between the groups. For the conditioning phase, a three-way repeated measures MANOVA will be conducted separately for each of the target mechanisms (expected relevance and attention). For these models, pre-exposure groups will serve as the between-subjects factor with stimulus and trial being the within-subject factors, and the analysis model will assess the main effects of each variable, as well as all-two-way and three-way interactions. Follow-up analyses depend on the nature of effects that are present (e.g., type of interactions or main effects). We expect that differences between pre-exposure groups will be greater—particularly at the early trials for stimulus CS^+^ _P_ —than for the other stimulus conditions. As such, follow-up analyses will focus on the degree to which means differ by pre-exposure group and stimulus type for each trial using a Bonferonni-adjusted alpha, as well as examining plots of the mean response by stimulus type and trial for each pre-exposure group.

#### Hypothesis 3

Higher pain sensitivity scores will be associated with increased engagement of the target mechanisms of expected relevance and attention.

To test the third hypothesis, individual differences in pain sensitivity will be included in the above analyses as a moderating variable. This will be done by testing interactions between pre-exposure and pain sensitivity.

#### Hypothesis

1b: A larger dose of pre-exposure, compared to a smaller dose, will strengthen the LI of conditioned fear acquisition, recall, and retention. Hypothesis 2b: Compared to pre-exposure with only the target stimulus, compound pre-exposure will strengthen the LI of conditioned fear acquisition, recall, and retention.

**Fig. 1:**
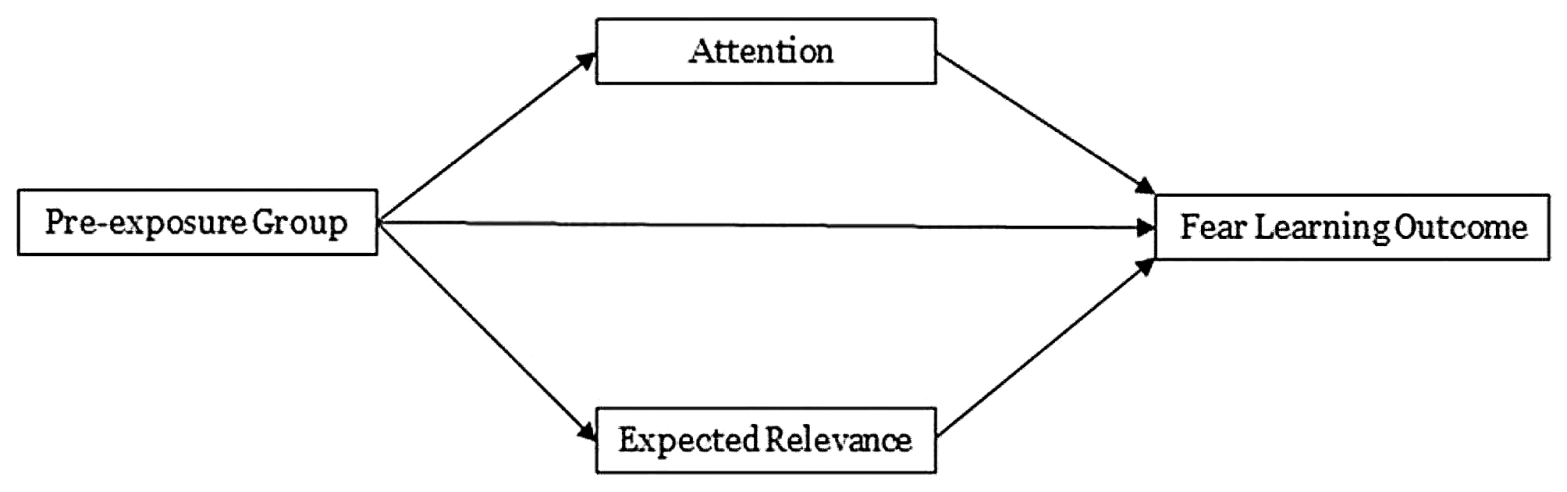
Conceptual display of the path model for latent inhibition

To assess hypotheses 1b and 2b, mediation analysis will be conducted to estimate direct, indirect, and total effects associated with the pre-exposure groups on the distal outcomes of conditioned fear acquisition, recall, and retention. Figure 1 shows the conceptual model that will be estimated for each distal outcome. In the parallel mediation model shown in Fig. 1, the pre-exposure groups are hypothesized to impact the mediators (expected relevance and attention), which in turn are hypothesized to affect a given fear outcome. Note that there are no within-subjects variables in this path model as the scores for the mediating variables will be obtained from the first trial of the conditioning phase, where stimulus group differences are expected to be strongest, and scores from a given distal outcome will be computed as the average of the scores across specific trials in the recall and then retention phase. Note that if the pre-exposure variable was found to interact with stimulus type in hypotheses 1a or 2a, then this model will be estimated separately for each stimulus type to assess if mediation is present for each stimulus.

Parameters for the mediation analyses will be estimated in Mplus with maximum likelihood estimation and percentile bootstrapping (with 20,000 bootstrap samples), with the latter used to test for the presence of indirect effects. Bootstrapping is a recommended method for mediation analysis given that indirect effects are non-normally distributed [39–41], as each indirect effect is computed as a *product* of parameter estimates.

## Discussion

Dental fear is a longstanding issue impacting oral health across the globe [3]. Associative conditioning is implicated as a major etiological factor for excessive dental fear [2], and LI from pre-exposure provides a potential strategy for disrupting dental fear learning. Currently, little is known about the mechanisms responsible for the LI of dental fear. This protocol provides an overview of one study in a series of studies aimed at identifying the mechanisms underlying the LI of dental fear. The primary goal is to assess whether higher pre-exposure dose and stimulus novelty (compound pre-exposure) potentiate the mechanisms of LI proposed in a prominent theoretical model by Hall and Rodríguez [18]. Based on this model, it is anticipated that both factors will engage the theorized mechanisms of expected relevance and attention, and thereby increase the LI of dental fear. To our knowledge, the study will be the first to experimentally assess the impact of compound pre-exposure and pre-exposure dose on the LI of dental fear. Moreover, a strength of this research is the inclusion of a second visit to assess fear recall, as many fear conditioning studies do not provide an extended assessment, which has direct relevance for intervention. Another strength is the inclusion of participants from a wide age range, 6 to 35 years old, which is also uncommon in fear conditioning experiments. This is notable, as age of first dental visits in some cases can extend into adulthood [42]. A final goal of the study is to assess if the mechanisms and indicators of LI of dental fear are associated with individual differences in pain sensitivity.

The results of the present experiment should provide practical information regarding necessary characteristics of future interventions. Specifically, if pre-exposure interventions are devised to diminish dental fear learning, it will be important to know whether such interventions will benefit from higher pre-exposure doses and if compound stimulus presentation will strengthen the effectiveness of an intervention. For example, if a larger pre-exposure dose potentiates LI, dental fear interventions would benefit from long pre-exposure sessions. Ultimately, experiments such as this will provide interventionists the data needed to devise effective strategies to help prevent the likelihood of dental phobia.

## Data Availability

No datasets were generated or analysed during the current study.

## Abbreviations

MANOVA: Multivariate analysis of variance
CS^−^: Unpaired control stimulus
CS^+^_NP_: Non-pre-exposed to-be conditioned stimulus
CS^+^_P_: Pre-exposed to-be conditioned stimulus
LI: Latent inhibition
ms: Millisecond
psi: Pounds per square inch
UCS: Unconditioned stimulus
